# Vulvar squamous cell carcinoma with sarcoma-like stroma: A case report and review of the literature

**DOI:** 10.1186/1746-1596-6-95

**Published:** 2011-10-01

**Authors:** Marco Petrillo, Giacomo Corrado, Arnaldo Carbone, Gabriella Macchia, Gabriella Ferrandina

**Affiliations:** 1 Department of Oncology, Gynaecologic Oncology Unit, Catholic University, Campobasso, Italy; 2 Department of Human Pathology, Catholic University, Campobasso, Italy; 3 Radiotherapy Unit, Catholic University, Campobasso, Italy

## Abstract

Vulvar squamous cell carcinoma with sarcoma-like stroma represents an extremely rare histological entity showing the co-existence of both epithelial and mesenchymal features: these tumors, firstly described in the skin by Martin and Stewart in 1935 have been further described in other anatomic sites including oral cavity, larynx, breast, lung and oesophagus. The complexity of the histology, as well as its aggressive clinical behaviour makes the diagnosis and the exploitment of effective therapeutic approaches very difficult, so that no definitive guidelines for treatments are currently available. Here, we describe a case of advanced stage vulvar squamous cell carcinoma with sarcoma-like stroma showing an unfavourable prognosis despite the use of an aggressive multimodal approach. A revision of the currently published cases have been also provided.

## Background

Vulvar malignancies are rare tumors accounting for almost 4% of all gynaecological cancer, and are still considered to be mostly a disease of older women [1]. While squamous cell carcinoma contributes approximately to 90% of vulvar tumors, mesenchymal neoplasias are uncommon, and typically show an aggressive clinical behaviour [1]. An extremely rare histological entity is represented by vulvar malignancies showing the co-existence of both epithelial and mesenchymal features: these tumors, firstly described in the skin by Martin and Stewart in 1935 have been further described in other anatomic sites including oral cavity, larynx, breast, lung and oesophagus [2,3]. The first case of vulvar squamous cell carcinoma showing the co-existence of areas with sarcomatoid features was reported in 1983 by Steeper et al [4]. Since then, few other cases have been published characterizing vulvar squamous cell carcinoma with sarcoma-like stroma (VSCS) as an aggressive disease typically associated with early development of both local recurrences and distant metastases [3]. The complexity of the histology, as well as the aggressive clinical behaviour makes the diagnosis and the exploitment of effective therapeutic approaches very difficult, so that no definitive guidelines for treatments of this malignancy are currently available.

Here, we describe a case of VSCS highlighting the diagnostic and clinical challenges in the context of the available literature.

## Case presentation

**Figure 1:**
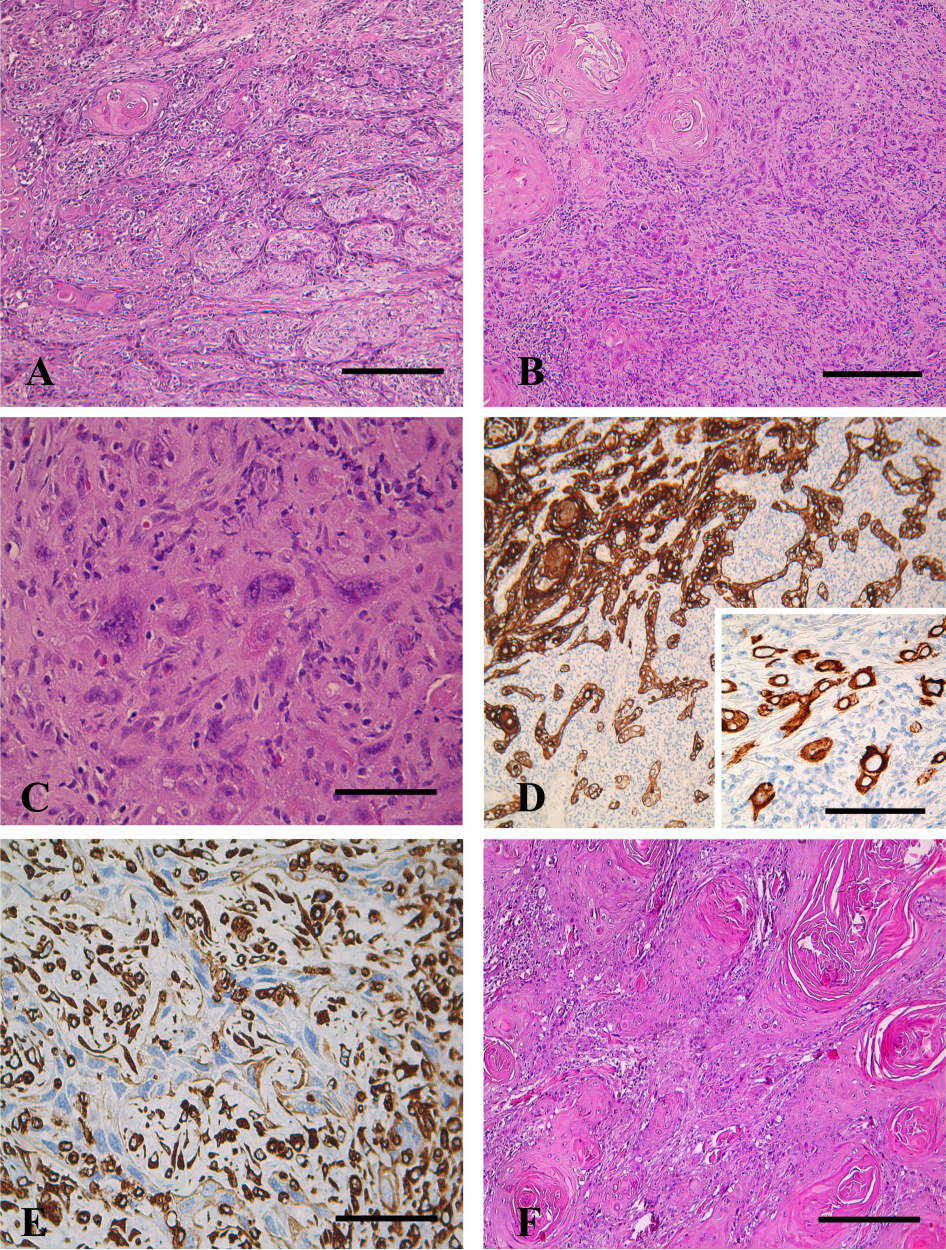
**Vulvar squamous cell carcinoma with sarcoma-like stroma (A, B, C, D, E tumor features at presentation site; F local tumor recurrence)**. **A, B**. Good squamous differentiation with keratinous pearls at tumor surface (upper-left corner in A and B) and less differentiated, spindle shaped cells in deeper portions of the tumor (right in A and B). Presence of scattered anaplastic, pseudosarcomatous cancer cells in a dense reactive stroma is shown in B (hematoxylin and eosin stain). **C**. At higher magnification ill defined cell borders are better seen (hematoxylin and eosin stain). **D**. Tumor cells presented positive for cytokeratins, more in the superficial, better differentiated portion of the tumor (upper-left corner). Inset shows the presence of reactivity for Cytokeratin in the isolated, giant neoplastic cells (Monoclonal Mouse Anti-Human Cytokeratin High Molecular Weight, clone 34βE12, DAKO, Carpinteria, CA, USA). **E**. Vimentin stain highlights a thick reactive vascular stroma, whereas giant and spindle shaped tumor cells appear negative (central portion of picture) (Dako, Carpinteria, CA, USA). **F**. Local tumor recurrence. Note the higher degree of tumor differentiation with abundance of keratinous pearls in this representative histological field (hematoxylin and eosin stain). Bars are 100 μm in A, B, D, F; 50 μm in E and D inset; 25 μm C.

In August 2009, a 79-year-old woman, 3 gravida 3 para, was admitted to the Gynaecologic Oncology Unit of the Catholic University of Campobasso, complaining of vulvar burning. Her family history did not reveal malignancies in first-degree relatives, and her past medical history was unremarkable. At gynaecological examination vagina, cervix and uterus appeared normal, whereas an ulcerated area (maximum diameter = 7 cm) involving the clitoris and both the right and left majus and minus labium was documented. Inguinal lymphadenopathies (maximum diameter = 1.5 cm) were bilaterally palpable. Biopsy of the lesion documented a well differentiated vulvar squamous cell carcinoma, and staging work-up, including chest X-rays, and abdominal CT scan, did not show any sign of distant sites of disease. Radical vulvectomy plus bilateral inguinal lymphadenectomy and vulvar reconstruction using the medial thigh VY advancement flap was performed. At histology, frank squamous maturation was particularly represented on tumor surface, whereas a gradient of dedifferentiation was observed toward deeper portions of tumor in which spindle shaped cells were more evident (Figure 1A, B, C). Both patterns were more or less represented in primary tumor (Figure 1A, B, C, D, E), as well as in lymph node metastases. Panel D and E also showed immunohistochemical analysis of high molecular weight cytokeratin (Monoclonal Mouse Anti-Human Cytokeratin High Molecular Weight, clone 34βE12, DAKO, Carpinteria, CA, USA) and vimentin (DAKO, Carpinteria, CA, USA) performed using a labeled streptavidin biotin peroxidase method (Visualization of the reaction was performed with the DAKO LSAB 2 kit peroxidase). Both squamous cell carcinoma and sarcomatoid components showed reactivity for high molecular weight cytokeratins, especially in the better differentiated areas (Figure 1D); vimentin highlighted the dense stromal reaction, whereas tumor cell resulted consistently negative (Figure 1E). Staining for HHF-35 (DAKO, Carpinteria, CA, USA) and S-100 (DAKO, Carpinteria, CA, USA) was also documented in areas with sarcomatoid features (data not shown). Considering the morphological features showing the presence of two easily identifiable epithelial and sarcomatoid components, the apparent transition from carcinomatous to sarcomatoid areas, as well as the results of the immunohistochemical analysis revealing reactivity of giant nucleated cells for cytokeratin with negative staining for vimentin, the case was finally defined as vulvar squamous cell carcinoma with sarcomatoid features (VSCS). Overall lymph node metastases were documented in 5 of 47 inguinal lymph nodes and final staging was pT2N2M0 according to TNM classification [5]. Surgical margins of resection appeared uninvolved. Given the occurrence of bilateral groin wound dehiscence requiring approximately 3 months of intensive wound care for complete resolution, the original treatment plan including chemotherapy plus radiation had to be shifted to systemic treatment: considering the paucity of data about medical treatment of this neoplasia, a regimen including platinum agents as well as anthracyclines was chosen given the widely recognized activity of these two classes of drugs in epithelial and sarcomatous neoplasia, respectively [2,3]. Considering also age and clinical conditions, the patient was triaged to the less toxic combination of carboplatin (AUC 5) and pegylated liposomal doxorubicin (30 mg/m^2^) q21. After completion of 6 cycles of primary treatment, the patient started the routinary follow-up program, and only 1 month after the last cycle gynaecological examination revealed the presence of a fixed nodule in the left majus labium (maximum diameter = 2.5 cm). Complete surgical excision of the suspected area was carried out and final histology documented the same phenotypic features of the primary tumor with squamous cell differentiation as the largely dominating component (Figure 1F). One month later, PET-computed tomography showed abnormal uptake of the radiotracer in the right lung hilar, mediastinal, and right obturatory lymph nodes. The patient was triaged to receive salvage chemotherapy with paclitaxel (135 mg/m^2^) q21 and consolidation by involved field stereotactic radiotherapy. Chest/abdominal CT scan was performed after 4 cycles of chemotherapy documenting a partial response to treatment with the disappearance of the right obturatory lymphadenopathies and a significant reduction of the mediastinal (5 mm vs 10 mm as maximum diameter), and right hilar (12 mm vs 19 mm as maximum diameter) lymphadenopathies. Thereafter, extracranial stereotactic radiotherapy was planned by the Precise-Plan treatment planning system. Patient was immobilized using the Stereotactic Body-Frame (Elekta) and a class solution with 4 non-coplanar fixed beams based on the tetrad configuration was used to treat pre-chemotherapy nodal targets. Five consecutive daily fractions were delivered to mediastinal and right hilar nodes up to a total dose of 45 Gy/9 Gy fraction and to right obturatory lymphadenopaties up to 40 Gy/8 Gy fraction. Treatment was very well tolerated and obtained a radiological complete response. Two months later, in January 2011, the patients was admitted to our Institution complaining of severe asthenia and moderate dyspnoea. Total body CT scan documented multiple bilateral pulmunary metastases (maximum diameter = 2 cm), enlarged right iliac lymph nodes (maximum diameter = 2 cm), a focal liver lesion (maximum diameter = 2 cm), and an ulcerated perineal area infiltrating the urethra (maximum diameter = 5 cm). The patient died after 1 month due to acute respiratory failure, after 18 months from initial diagnosis.

## Discussion

The histopathological diagnosis of VSCS is usually formulated in presence of two separate components, one with clinical squamous features generally located on the tumor surface, and the other one located in the deeper portions of the tumor characterized by anaplastic sarcomatoid morphology. Differential diagnosis should also include vulvar sarcoma, malignant fibrous histiocytoma, and amelanocitic malignant melanoma [4]. However, along the diagnostic process, the most difficult problem is certainly represented by distinguishing VSCS from malignant mixed Müllerian tumor (MMMT). In this context, it should be taken into account that the most common location of MMMT is represented by uterine corpus followed by cervix and vagina, so that the possibility that vulvar disease could be just a metastatic site from primary uterine tumor has to be not underestimated. Moreover, the epithelial component of MMMT is usually represented by adenocarcinoma rather than squamous cells, and is usually intermingled with the sarcomatous components rather than showing an easily visible division into superficial versus deeper areas as in VSCS; another morphological VSCS feature not shared by MMMT is the apparent transition from carcinomatous to sarcomatoid areas [4]. Although these peculiar morphological features may be helpful in identifying VSCS, the formulation of an appropriate diagnosis on the basis of conventional hematoxylin and eosin stain would be difficult. In this context, the presence of diffuse immunoreactivity for cytokeratin in spindle shaped tumor cells have been very useful to rule out a mesenchymal tumor which usually does not show a positive staining for cytokeratin. However, it should be taken into account that some mesenchymal malignancies such as Ewing's sarcoma and epithelioid sarcoma may express cytokeratin so that it can be helpful to utilize a panel of antibodies, including anti-vimentin usually expressed in sarcoma cells. Finally, in our case, as in the one reported by Parham et al. [6], the initial surgical sampling did not allow to draw a definitive diagnosis, thus suggesting that an extensive tissue sampling is necessary to formulate a correct diagnosis of this rare entity.

The histogenesis of VSCS remains to be clarified, but it is more commonly believed that VSCS might arise from a metaplastic process of the carcinomatous component. In this context, our results showing reactivity of both squamous cell carcinoma and sarcomatoid component for high molecular weight cytokeratin seem to suggest the epithelial origin of the sarcoma-like cells, as proposed by other authors [3,7]. It remains to be verified whether this phenomenon could in term hesitate into the occurrence of what is called epithelial mesenchymal transition.

The comparison with other similar cases reported in the literature (Table 1) is difficult due to the paucity of series, and the wide heterogeneity of cases relative to the original definition of histology, and also stage at presentation, and treatment modalities; as far as clinical outcome is concerned, with the exception of some cases diagnosed at early stage disease [3,6] or characterized by a bizarre continuously changing morphology at recurrence [6], the vast majority of cases experienced a rapidly progressive and fatal disease [4,6-12], despite the exploitment of multimodal treatment with radical surgery and adjuvant radiotherapy and/or chemotherapy [12,13].

**Table 1:** Clinico-pathological characteristics, treatment details, and follow-up status of the previously published cases of sarcomatoid squamous cell carcinoma of the vulva.

Author	No. cases	Age	FIGO Stage	Primary treatment	Pathological Node Status	Histologic Report	DFS	Overall Survival
Way [8]	6	-	-	Surgery	n.a.	*Unusual epithelioma*	-	Less than 4.5 years

Gosling [9]	2	-	-	Surgery	n.a	*Spindled squamous carcinoma*	-	-

Copas [13]	1	54	III	Radical vulvectomy with bilateral groin and pelvic lymphadenectomy followed by adjuvant CT and RT	+	*Poorly differentiated squamous cell carcinoma*	1 month	2-3 months

Steeper [4]	1	89	-	RT followed by simple vulvectomy	n.a.	*Pseudosarcomatous squamous cell carcinoma*	8 months	32 months

LiVolsi [10]	2	-	-	n.a.	n.a	*Carcinoma with sarcomatoid features*	-	-

Santeusanio [7]	1	77	IV	Radical vulvectomy with bilateral inguinal lymphadenectomy	+	*Carcinoma with sarcoma-like features*	15 days	1 month

Parham [6]	1	54	I	Local excision	n.a.	*Mixed soft tissue sarcoma with atypical squamous cell*	3 years	More than 6 years

Cooper [12]	1	73	III	Radical vulvectomy with bilateral inguinal lymphadenectomy	+	*Sarcomatoid squamous cell carcinoma*	5 months	-

Choi [3]	1	43	II	Local excision with bilateral inguinal lymphadenectomy	-	*Sarcomatoid squamous cell carcinoma*	Non evident disease	More than 2 years

Loizzi [11]	1	85	II	Radical vulvectomy with center inguinal lymphadenectomy	-	*Carcinosarcoma*	1 month	2 months

Present case	1	79	III	Radical vulvectomy with bilateral inguinal lymphadenectomy followed by CT	+	*Sarcomatoid squamous cell carcinoma*	1 month	18 months

CT: chemotherapy; RT: radiation therapy

Even recognizing that VSCS might have an intrinsic biological aggressiveness [7], the observation that cases presenting at diagnosis with early stage disease [3,6] may experience a very long survival, emphasize the need to make any effort in order to achieve an early diagnosis.

As already acknowledged, no specific guidelines for treatment of VSCS are available, due to its rare occurrence. Radical surgery seems to guarantee in some cases an adequate local control, whereas the role of adjuvant chemotherapy remains unclear: in our case, a combination regimen with platinum and anthracyclines, which are recognized to be active against carcinomatous and sarcomatous component [14], resulted unsuccessful with rapid development of local recurrence. On the other hand, we reported a partial response to taxanes and even with the disappearance of some sites of metastatic disease, thus expanding the range of drugs potentially available against this disease. Radiation therapy was explored as primary treatment [4], as in the adjuvant setting [13] with unsatisfying results: however, in our case disappearance of all metastatic sites was achieved administering stereotactic radiotherapy [15], suggesting that, at least in principle these tumors may retain sensitivity to radiotherapy, especially if administered by hypofractionation modality (high dose/fraction in a short overall treatment time) and stereotactic technique. Overall, our case considering the advanced stage of disease, showed a relatively longer OS and also an acceptable symptom-free period compared to results from the available literature, probably due to the use of a multimodal therapeutic approach.

## Conclusions

The rarity of this entity, the diagnostic difficulties, and poor survival highlights the need for a systematic collection of these tumors in order to help pathologists and gynaecologists to achieve an early diagnosis, and develop in the next future more effective therapeutic strategies. In this context, the biological characterization of this rare neoplasia seems worth while.

## Consent

Written informed consent was obtained from the patient for publication of this case report and any accompanying images.

A copy of the written consent is available for review by the Editor-in-Chief of this journal.

## Competing interests

The authors declare that they have no competing interests.

## Authors' contributions

MP conceived the study, and drafted the final version of the manuscript. GC participated in manuscript drafting. AC carried out histopathological evaluation and helped in drafting the manuscript. GM participated in manuscript drafting. GF conceived of the study, and participated in its design and coordination and helped to draft the manuscript. All authors read and approved the final manuscript.
